# Identifying Clinically and Functionally Distinct Groups Among Healthy Controls and First Episode Psychosis Patients by Clustering on EEG Patterns

**DOI:** 10.3389/fpsyt.2020.541659

**Published:** 2020-09-18

**Authors:** Xiaodong Qu, Saran Liukasemsarn, Jingxuan Tu, Amy Higgins, Timothy J. Hickey, Mei-Hua Hall

**Affiliations:** ^1^ Department of Computer Science, Brandeis University, Waltham, MA, United States; ^2^ Psychosis Neurobiology Laboratory, McLean Hospital, Harvard Medical School, Belmont, MA, United States; ^3^ Schizophrenia and Bipolar Disorders Program, McLean Hospital, Belmont, MA, United States

**Keywords:** mismatch negativity, first episode psychosis, heterogeneity, machine learning, K-means clustering, longitudinal study, low frequency

## Abstract

**Objective:**

The mismatch negativity (MMN) is considered as a promising biomarker that can inform future therapeutic studies. However, there is a large variability among patients with first episode psychosis (FEP). Also, most studies report a single electrode site and on comparing case–control group differences. Few have taken advantage of the full wealth of multi-channel EEG signals to examine observable patterns. None, to our knowledge, have used machine learning (ML) approaches to investigate neurophysiological derived subgroups with distinct cognitive and functional outcome characteristics. In this study, we applied ML to empirically stratify individuals into homogeneous subgroups based on multi-channel MMN data. We then characterized the functional, cognitive, and clinical profiles of these neurobiologically derived subgroups. We also explored the underlying low frequency range responses (delta, theta, alpha) during MMN.

**Methods:**

Clinical, neurocognitive, functioning data of 33 healthy controls and 20 FEP patients were collected. 90% of the patients had 6-month follow-up data. Neurocognition, social cognition, and functioning measures were assessed using the NCCB Cognitive Battery, the Awareness of Social Inference Test, UCSD Performance-Based Skills Assessment, and Multnomah Community Ability Scale. Symptom severity was collected using the PANSS. MMN amplitude and single-trial derived low frequency activity across 24 frontocentral channels were used as main variables in the ML k-means clustering analyses.

**Results:**

We found a consistent pattern of two distinctive subgroups. We labeled them as “better functioning” and “poorer functioning” clusters, respectively. Each subgroup can be mapped onto either better or poorer clinical, cognitive, and functioning profiles. Also, we identified two subgroups of patients: one showed improved MMN and one showed worsening of MMN over time. Patients with improved MMN had better follow-up clinical, cognitive, and functioning profile than those with worsening MMN. Among the low frequency bands, delta frequency appeared to be the most relevant to the observed MMN responses in all individuals. However, higher delta responses were not necessarily associated with a better functioning profile, suggesting that delta frequency alone may not be useful in clinical characterization.

**Conclusions:**

The ML approach could be a robust tool to explore heterogeneity and facilitate the identification of neurobiological homogeneous subgroups in FEP.

## Introduction

Psychosis is one of the most disabling conditions worldwide (1, 2). Early intervention can play a substantial role in improving long-term outcomes, although there is a large variability in treatment responsiveness in the first episode of psychosis (FEP) patients (3). FEP patients demonstrate a wide range of cognitive and neurophysiological impairments and are considerably heterogeneous in the functional outcome trajectories (4–6). Progress is undoubtedly hampered by considerable biological and clinical heterogeneity across FEP: effective treatments are unlikely to advance substantially until disease mechanisms are better understood, and biologically-based objective markers are available to tag the cardinal dysfunction, not the diagnoses or symptoms.

Detecting unexpected stimuli in the environment is a critical function of the auditory system. The mismatch negativity (MMN) is the best-studied electrophysiological marker of deviance detection. It is typically elicited in oddball paradigms and measured as the difference between the event-related potential (ERP) responses to deviant (*e.g.*, duration, pitch) and standard stimuli. The magnitude of MMN ERP is typically greater in the frontocentral electrode sites and peaks between 100 and 250 ms (7, 8). Larger responses (more negative) to rare/deviant stimuli are thought to represent the detection of regularity violation (9–11). Such predominantly automatic (preattentive) process of detecting a “mismatch” between the deviant stimulus and a sensory memory trace might be a critical transitional step from sensory-based processing to the subsequent engagement of higher attentional neural networks necessary for cognitive and psychosocial functioning (12–16).

MMN, particularly the duration-deviant MMN (dMMN), is considered as a promising candidate biomarker that can inform future therapeutic studies of FEP (17–22). Evidence suggests that MMN peak amplitude is a sensitive index of NMDA (15, 23–25) and nicotinic receptor functioning (26). In healthy and chronic schizophrenia (SZ) patients, MMN activity is significantly correlated with distinct domains of cognitive (27–29) and psychosocial, work functioning, and independent living functioning (17, 30–32). Furthermore, MMN deficit increases with the progression of the disease. Meta-analysis found that in FEP patients, the effect size of dMMN deficit is small to medium (Cohen’s d = 0.47) (20); in chronic schizophrenia patients, a systematic increase in effect size was found as a function of illness duration, indicating that dMMN deficit reflects, to some extent, disease progression (33, 34). Although dMMN deficit is reported in FEP patients, the small effect size suggests a large variability among patients. Given that heterogeneity is a key feature of FEP that manifests on clinical, neurobiological, and functioning levels resulting in a substantial barrier to understand disease mechanisms, a data driven approach to stratify FEP patients into homogeneous subgroups would allow a better understanding of the source of variability and biological mechanisms.

Moreover, the relationships between dMMN change over time and its corresponding clinical changes are largely unknown. Longitudinal MMN studies in FEP are rare. Pitch-deviant MMN studies showed that FEP patients with the most impaired MMN amplitudes at baseline showed the most severe disability at follow-up (35) and that MMN was intact at baseline in a majority of FEP patients but worsened at follow-up (36). Identifying subgroups of FEP patients with distinct patterns of dMMN changes over time as well as the corresponding clinical, cognitive, or functioning changes may facilitate early identification of a subgroup of patients at heightened risk for cognitive and functioning decline.

The majority of clinical studies of MMN typically focus on using a single electrode site (Fz) and on comparing case–control group differences. Few have taken advantage of the full wealth of information about brain dynamic processes and observable patterns contained in multi-channel MMN EEG signals. Conventional approaches based on group (case–control) comparisons assume controls and patients as homogeneous populations which does not adequately address the heterogeneity of within-group individual differences. Machine learning (ML) data-driven approaches to address heterogeneity have received renewed interest in partitioning individuals into more homogeneous subgroups (37, 38). The advances in ML approaches make it possible to extract information from complex and high-dimensional data.

In terms of exploring the underlying EEG frequency responses during MMN, previous studies showed that MMN was primarily comprised of lower range frequency evoked oscillations including delta, theta, and alpha (39–42). In controls, theta–alpha frequency was found to be the most significant contributor for MMN, while in patients with SZ spectrum disorders, delta range activities were found to explain the most variance of observed MMN abnormalities. MMN reflects activity primarily in low frequency band, which is thought to depend primarily upon interplay between cortical pyramidal neurons and somatostatin type local circuit GABAergic interneurons (42). However, few studies have examined patients with SZ (41). It is currently unknown to what extent the theta frequency activity plays a role in the generation of MMN in FEP patients. Moreover, it remains unclear to what extent the low frequency in the delta, theta, and alpha range activity is related to cognitive or daily functioning measures; that is, whether abnormal low frequency activity in the delta, theta, and alpha bands is correlated with impaired cognitive or functional measures with respect to the correlations found using MMN amplitude. Finally, the longitudinal change of low frequency range activity in FEP has not been reported in the literature.

In this study, we collected an array of functional, cognitive, clinical, and multi-channel MMN data from a cohort of controls and FEP patients (90% had 6-month follow-up data). We aimed to use an unsupervised ML clustering technique to address several key research questions: i) to stratify individuals into more homogeneous subgroups based on multi-channel MMN activities and examine whether each neurobiologically derived subgroup could be mapped onto a consistent pattern of functional, cognitive, and clinical profile; ii) to stratify FEP patients into distinct clusters based on the change of dMMN over a 6-month period and examine whether patient’s MMN change in each cluster could correspond to a consistent pattern of follow-up functional, cognitive, and clinical profile; iii) to investigate the magnitude of low-frequency (delta, theta, alpha) activities in both controls and patients; iv) to stratify individuals into more homogeneous subgroups based on multi-channel low frequency activity and examine whether delta, theta, and alpha range activities are related to cognitive or daily functioning measures.

We applied data driven strategy, K-means, to first addressed heterogeneity in MMN and low frequency measures among patients and controls in a longitudinal study. ML algorithm empirically classifies individuals based on mathematical calculations of individual’s multimodal MMN or each delta, theta, or alpha activities. We then performed analyses to characterize the cognitive, symptom severity, and functioning performances of the empirically derived clusters to address the question whether data driven classification results were clinically meaningful.

## Methods

### Participants

Demographics, MMN, cognition (NCCB MATRICS Consensus Cognitive Battery), and clinical data of 33 healthy controls and 20 FEP patients were collected. Eighteen of the FEP patients also had 6-month follow-up data (**Table 1**). Patients were identified and recruited for the study within 12 months of first episode of psychosis. After enrollment, clinical diagnostic interview and the series of tests including EEG and neurocognitive tests were administered within 45 days. Each subject was assessed by the Structured Clinical Interview for DSM-IV (SCID). Patients were clinically stable. Study inclusion criteria were: 1) age between 18 and 45 years; 2) fluency in English; 3) IQ > 70; 4) patients with FEP diagnosed with SZ, schizoaffective disorder, schizophreniform disorder, psychotic disorder not otherwise specified, or psychotic bipolar disorder. Exclusion criteria consisted of: 1) diagnosed neurological disorder; 2) brain injury including stroke or serious head injury resulting in loss of consciousness; 4) hearing impairments, blindness, or deafness; 5) electroconvulsive therapy within the past 6 months; 6) outside the age range of 18–45 years. HC subjects were recruited from the Partners Research Portal and subject to the same exclusion criteria plus the following: no current or past history of psychotic or affective disorders, no substance abuse or previous chronic dependence, and no first-degree relative with a history of psychosis or bipolar disorder. Patients with substance abuse or dependence within 6 months were excluded. As a history or lifetime diagnosis of substance abuse or dependence is common among patients, FEP patients with previous substance abuse history were not an exclusion. Similarly, depression is common in the general population (~20–25%), healthy controls having relatives with a history of depression were not an exclusion. The study was approved by the McLean Hospital Institutional Review Board. All subjects provided written informed consent after receiving a complete description of the study.

**Table 1:** Comparisons between controls, baseline patients, and 6-month follow-up patients.

Variables	Controls (N=33)	Baseline Patients (N=20)	6m Follow-up Patients (N=18)	Statistics P value
	Mean (Std Errors)	Mean (Std Errors)	Mean (Std Errors)	
Age	22.91 (3.9)	22.7 (3.2)	23.39 (3.3)	F = 0.19 p = 0.83
Females (count, %)	12 (36.36%)	7 (35.00%)	6 (33.33%)	χ = 0.05 p = 0.98
Education (years)	15.55 (1.7)	14.95 (1.6)	15.06 (1.6)	F = 0.97 p = 0.38ß
UPSA total score	83.45 (8.3)	79.99 (10.9)	82.52 (12.0)	F = 0.58 p = 0.56
MCAS total score	54.75 (0.6)	48.1 (5.8)	48.0 (6.2)	F = 17.38 p <0.0001
MATRICS Neurocognitive Composite Score	50.45 (5.2)	46.21 (6.4)	48.63 (8.1)	F = 2.70 p = 0.07
MATRICS Social Subscore	54.52 (6.6)	53.58 (11.5)	55.33 (13.8)	F = 0.13 p = 0.88
TASIT	55.77 (4.5)	53.69 (6.4)	54.67 (5.2)	F = 0.579 p = 0.46
PANSS positive	N/A	14.45 (6.8)	13.18 (5.4)	t = 0.62 p = 0.27
PANSS negative	N/A	12.5 (3.8)	10.41 (3.5)	t = 1.70 p = 0.048
PANSS general	N/A	30.6 (7.9)	26.70 (8.4)	t = 1.45 p = 0.08
PANSS total	N/A	57.55 (16.7)	50.29 (16.1)	t = 1.33 p = 0.09
Chlorpromazine equivalents	N/A	226.51 (234.3)	292.45 (241.6)	t = -0.74 p = 0.77

Means with standard deviations in parentheses unless specified otherwise; UPSA, UCSD Performance-based Skills Assessment; MCAS, Multnomah Community Ability Scale; MATRICS, Measurement and Treatment Research to Improve Cognition in Schizophrenia; TASIT, The Awareness of Social Inference Test; PANSS, Positive and Negative Syndrome Scale; CPZ, chlorpromazine equivalents.

### Neurocognitive, Functioning, and Clinical Assessments

Neurocognition was assessed using the MATRICS Consensus Cognitive Battery [MCCB; (43)]. A MCCB neurocognitive composite score (the average of all MATRICS tasks excluding the social cognition MSCEIT task) was calculated. Social Cognition was assessed using two measures: the Awareness of Social Inference Test (TASIT)—Part Two (44, 45) which measures social inference/Theory of Mind, and the MSCEIT from the MCCB, which measures social and emotional reasoning.

Functional capacity was assessed using the UCSD Performance-Based Skills Assessment, Brief (UPSA-B) (46, 47). The UPSA-B is a performance-based measure designed to evaluate participants’ abilities to perform everyday tasks considered necessary for independent functioning in the community. Total scores range from 0 to 100 points; higher scores reflect better performance. Community functioning was evaluated using an abbreviated version of the Multnomah Community Ability Scale (MCAS) (48), an interview-based measure developed for assessment of community outcomes in psychiatric populations. This brief version probes several aspects of community functioning including independence in daily living, instrumental role functioning, and social interest and engagement (49–51). Clinical assessment was performed using the Positive and Negative Syndrome Scale (PANSS) subscales for Positive, Negative, and General symptoms (52).

### EEG Procedures and Data Processing

The electroencephalogram (EEG) was recorded continuously using the BioSemi Active Two system (BioSemi Inc, Amsterdam, Netherlands) at a digitization rate of 512 Hz, with a bandpass of DC–104 Hz, and a Common Mode Sense (CMS) as the reference (PO2 site) using a 64-channel electrode cap. EOG electrodes were placed below and at the outer canthi of the left eye. A duration MMN paradigm was used to elicit MMN. Stimuli consisted of 1,200 trials presented to the subjects through foam insert earphones. 85% of the stimuli were standard [S1] tones (1,000 Hz, 100 ms), and 15% were duration deviant [S2] tones (1,000 Hz, 150 ms) with an inter-stimulus interval of 200 ms. Participants were instructed to watch a silent cartoon/video clip (BBC natural program or Charles Brown) during the stimulus presentation.

**Figure 1:**
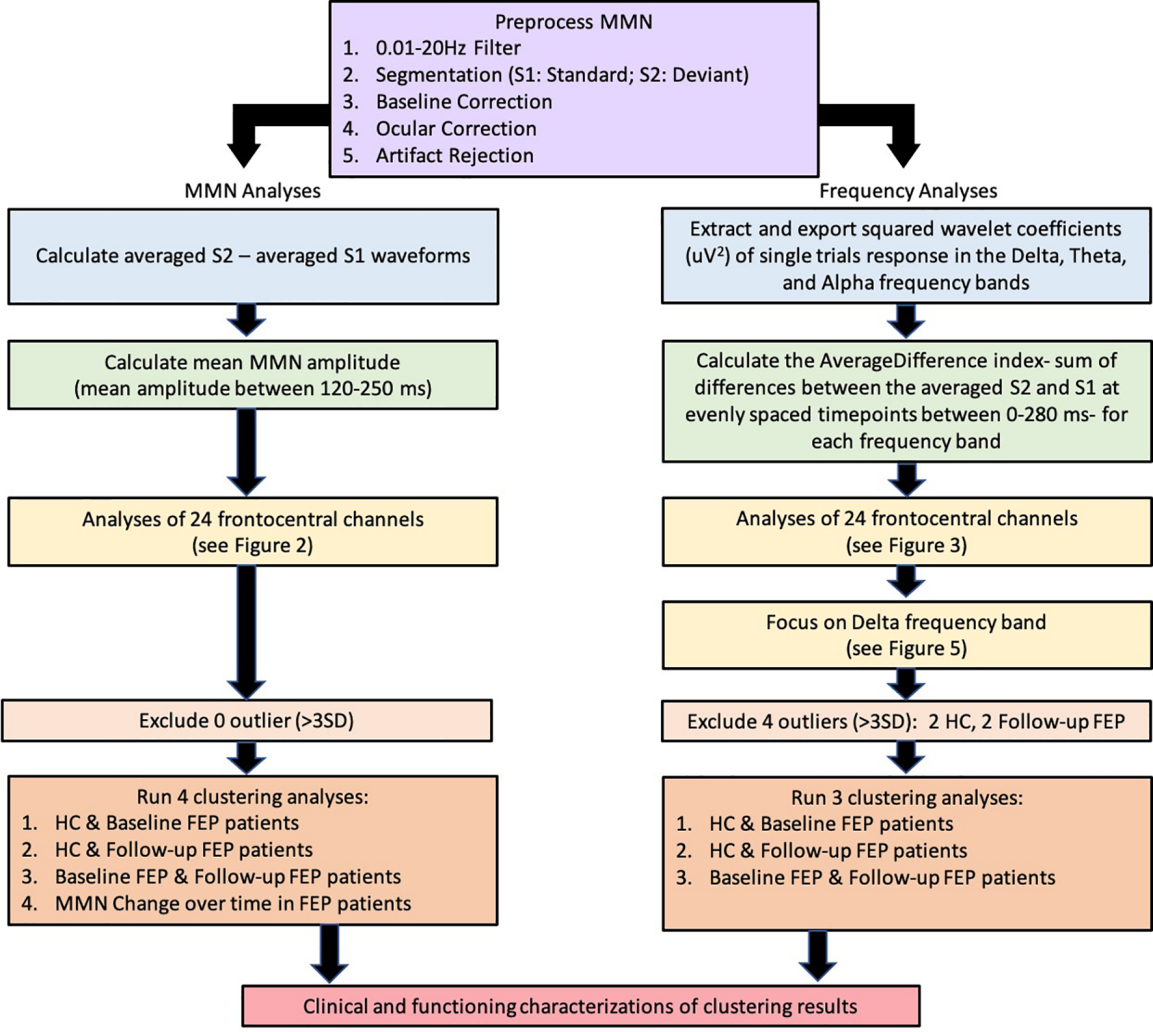
Data processing and analysis pipelines.

Data processing and analysis pipelines are presented in **Figure 1** Flow Chart. Data processing was performed offline using Brain Vision Analyzer 2 (Brain Products GmbH, Munich, Germany) and MATLAB R2017b (The MathWorks, Massachusetts, USA) and blind to group membership using automated procedures. Signals were re-referenced to an average of the mastoids and bandpass filtered between 0.01 and 20 Hz. Data were segmented by stimulus marker from −100 to 400 ms for MMN analysis and from −100 to 280 ms for frequency analysis. Segments were baseline corrected using −100 to 0 ms pre-stimulus time and eye-blink corrected using established measures (53). Artifact rejection for individual channels was performed and a given segment was rejected if the voltage gradient exceeded 50 μV/ms, amplitude was +/−100 μV, or the signal was flat (<0.5 μV for >100 ms).

MMN waveforms were generated by subtracting standard (S1) from the deviant (S2) waveforms and the MMN amplitude was calculated as the mean amplitude (uV) between the time window of 120 to 250 s. Event-related low frequency measures were computed from the single-trial segments. Single-trial S1 and S2 segments were extracted after artifact rejection procedure using the Morlet wavelet transformation (squared wavelet transformation (uV^2^) for delta: 1–4 Hz; theta: 4–8 Hz; alpha: 7.5–13 Hz). Originally, there were a total of 1,020 S1 and 180 S2 segments. Artifact rejection procedure and an additional step for removing bad intervals led to having approximately 981 S1 segments and 172 S2 segments for each subject per frequency band.

For each frequency band, we computed the sum of the average difference (AverageDifference) index to capture the overall differences between the averaged S2 and S1 segments in each of the 24 channels (see equation below). First we computed the average of S2 (AvgS2) and S1 (AvgS1) in each channel ‘e’. C_S2_ is the number of S2 segments and C_S1_ is the number of S1 segments (*e.g*., C_S1_ = 981 for S1 segments *vs.* C_S2_ = 172 for S2 segments). Then AverageDifference is computed by summing up the difference between AvgS2 and AvgS1 over time ‘t’, starting from the stimulus onset (0 ms) to the end of the segment (280 ms).

The AverageDifference for each channel e is defined as the following:

$$AverageDifference_{e}=\Sigma_{t=time}(AvgS2_{e}^{\left(t\right)}-AvgS1_{e}^{\left(t\right)})$$

where:

$$AvgS1=\frac{\Sigma_{i}^{C_{S1}}S1_{i}}{C_{S1}}$$

$$AvgS2=\frac{\Sigma_{i}^{C_{S2}}S2_{i}}{C_{S2}}$$

Since AverageDifference was derived from squared wavelet transformed values and the summation of differences between AvgS2 and AvgS1, it is susceptible to extreme values which can significantly affect the clustering results. For instance, clustering a sample with one extreme value into two clusters can cause one cluster to contain only one subject while the other cluster contains the rest. To identify extreme outliers, we applied Median Absolute Deviation (MAD) procedure on the sum of the 24 channels, considered any observation with the AverageDifference value over three deviations away from the median to be an “outlier,” and removed such observations from clustering analyses. This procedure removed a total of four observations (two controls and two follow-up FEP patients).

**Figure 2:**
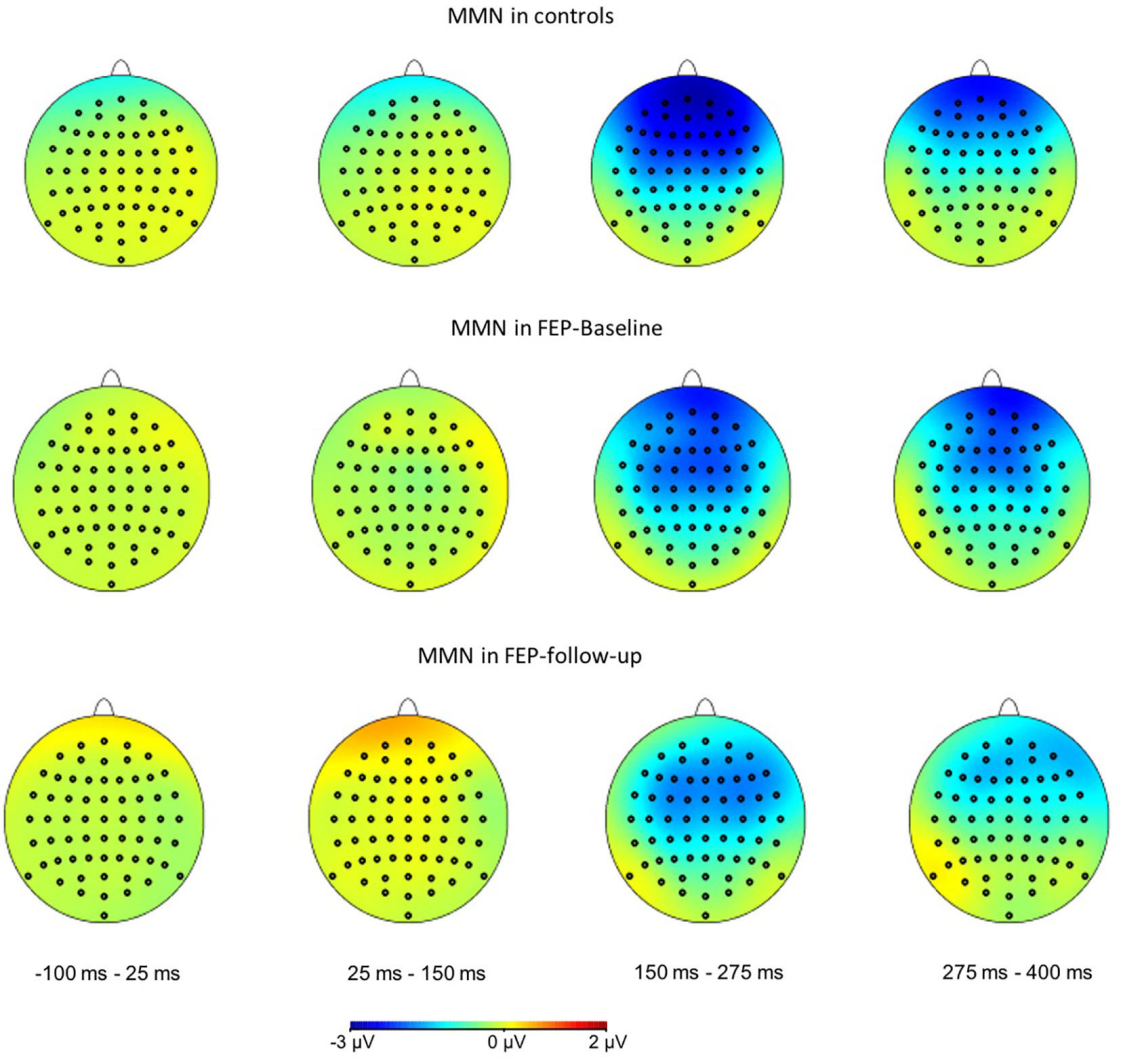
Scalp voltage topography maps, showing grand average of MMN amplitudes in controls (top), FEP patients at baseline (middle), and FEP patients at 6-month follow-up (bottom), across latency window from −100 to 400 ms. The frontal and central channels showed the strongest MMN responses.

**Figure 3:**
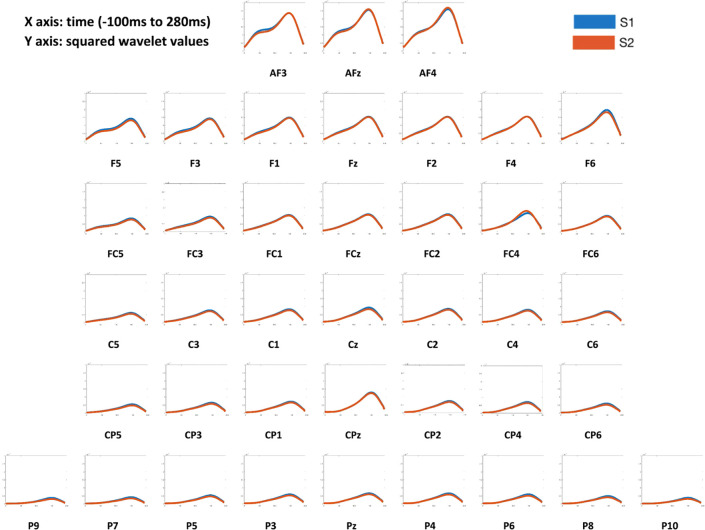
Averaged S1 and S2 responses across 40 channels of all subjects. X axis: time (−100 ms to 280ms). Y axis: squared wavelet values. In all participants, the frontal and central channels showed the greater averaged S1 and S2 response patterns than parietal channels.

The patterns of event-related responses from both MMN ERP (**Figure 2**) and averaged S1 and S2 activities (**Figure 3**) showed greater EEG signals at the frontal and central electrode sites than at the parietal sites, consistent with the literature (17). Therefore, data from 24 frontocentral sites (AFz, AF3, AF4, Fz, F1, F2, F3, F4, F5, F6, FCz, FC1, FC2, FC3, FC4, FC5, FC6, Cz, C1, C2, C3, C4, C5, C6) were used in the subsequent analyses.

### Cluster Analysis

**Figure 4:**
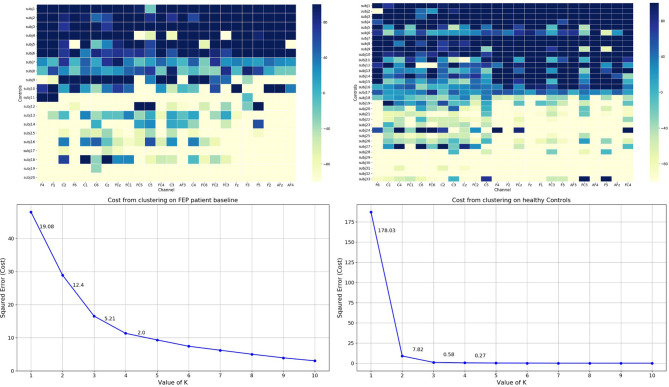
Top: Grand average of individual’s AverageDifference of delta frequency in healthy Control (Right) and FEP patient baseline (Left) across 24 channels. X axis: electrode sites; Y axis (far left): individual participants; Y axis (far right): the magnitude of the AverageDifference values. Bottom: The elbow method plots. X-axis: number of clusters; Y-axis: error/cost function. The cost (error) value drop is shown for each cluster solution.

To stratify subjects into more homogeneous subgroups, each individual’s MMN amplitudes and AverageDifference values across 24 channels were used to derive cluster assignments. The k-means algorithm was used. We applied the elbow method to empirically estimated the optimal number of clusters (54, 55). The elbow method calculates the cost function J for each of the cluster numbers (*e.g.*, from 1 to 10) by minimizing the error. The steeper drop of cost function (error) the better modelling of the data. The empirical elbow method indicates that there were two distinct clusters (see **Figure 4** and **Supplementary Figures S1** and **S2**).

#### Clustering Analyses Using MMN Amplitude Over 24 Channels

Because this study includes controls and a patient cohort with longitudinal follow-up data, four sets of analyses were run, clustering among (i) controls and patients at baseline, (ii) controls and patients at follow-up, (iii) patients at baseline and follow-up, and (iv) patients’ MMN changes over 6-month period (*i.e.*, follow-up MMN amplitude minus baseline MMN amplitude at each electrode channel). Once clusters were determined at each run, the resulting cluster assignments were mapped onto individuals’ clinical, cognitive, and functioning performances, including real-life functioning (UPSA, MCAS), neurocognition, social cognition (TASIT, MSCEIT or MATRICS Social Subscore), and symptom severity (PANSS), in order to characterize each cluster profile (**Figure 1**).

#### Clustering Analyses Using AverageDifference Over 24 Channels

Three separate clustering analyses were run based on the AverageDifference index, clustering among (i) controls and patients at baseline (ii) controls and patients at follow-up, and (iii) patients at baseline and patients at follow-up. Once clusters were determined at each run, the resulting cluster assignments were mapped onto individuals’ clinical, cognitive, and functioning performances, as described earlier.

## Results

### Clustering Analyses Using MMN Amplitude

Clustering results using MMN amplitude over 24 frontocentral channels were shown in **Table 2A**. In all three sets of clustering analyses (HC & baseline FEP; HC & follow-up FEP; baseline & follow-up FEP), the MMN amplitudes across all 24 electrode sites were larger (*i.e.*, more negative) in Cluster 1 than those in Cluster 2. In each channel differences between cluster 1 and 2 were significant at p < 0.05.

Table 2Clustering results of MMN amplitude and the clinical, cognitive, and functional characterizations of clusters.

**Table 2A:** Clustering results of MMN over 24 frontocentral channels

Electrode sites	HC & Baseline FEP		HC & Follow-Up FEP		Baseline & Follow-Up FEP	
	Cluster 1: Better functioning (N = 16)	Cluster 2: Poorer functioning (N = 37)	Cluster 1: Better functioning (N = 40)	Cluster 2: Poorer functioning (N = 11)	Cluster 1: Better functioning (N = 27)	Cluster 2: Poorer functioning (N = 11)
AFz	**-3.71 (1.1)**	**-1.48 (1.1)**	**-2.57 (1.3)**	**0.22 (1.3)**	**-2.26 (1.5)**	**0.09 (1.5)**
AF3	**-3.36 (1.3)**	**-1.36 (1.3)**	**-2.57 (1.2)**	**0.47 (1.2)**	**-2.19 (1.2)**	**0.34 (1.2)**
AF4	**-3.69 (1.2)**	**-1.51 (1.2)**	**-2.66 (1.3)**	**0.26 (1.3)**	**-2.4 (1.2)**	**0.07 (1.2)**
Fz	**-3.51 (0.8)**	**-1.44 (0.8)**	**-2.42 (1.0)**	**-0.27 (1.0)**	**-2.35 (0.9)**	**-0.32 (0.9)**
F1	**-3.51 (0.8)**	**-1.5 (0.8)**	**-2.5 (1.0)**	**-0.25 (1.0)**	**-2.38 (0.8)**	**-0.18 (0.8)**
F2	**-3.49 (0.9)**	**-1.56 (0.9)**	**-2.51 (1.0)**	**-0.14 (1.0)**	**-2.48 (0.8)**	**-0.13 (0.8)**
F3	**-3.43 (0.9)**	**-1.58 (0.9)**	**-2.58 (1.0)**	**-0.2 (1.0)**	**-2.41 (1.0)**	**-0.05 (1.0)**
F4	**-3.53 (0.9)**	**-1.39 (0.9)**	**-2.56 (1.1)**	**-0.17 (1.1)**	**-2.36 (0.8)**	**-0.19 (0.8)**
F5	**-2.95 (1.2)**	**-1.35 (1.2)**	**-2.24 (1.0)**	**0.06 (1.0)**	**-2.14 (1.2)**	**0.14 (1.2)**
F6	**-3.07 (0.9)**	**-1.4 (0.9)**	**-2.39 (1.0)**	**0.03 (1.0)**	**-2.3 (1.0)**	**-0.13 (1.0)**
FCz	**-3.28 (0.8)**	**-1.45 (0.8)**	**-2.31 (0.9)**	**-0.17 (0.9)**	**-2.42 (0.6)**	**-0.32 (0.6)**
FC1	**-3.44 (0.9)**	**-1.47 (0.9)**	**-2.43 (1.0)**	**-0.19 (1.0)**	**-2.45 (0.7)**	**-0.39 (0.7)**
FC2	**-3.41 (0.8)**	**-1.42 (0.8)**	**-2.37 (1.0)**	**-0.12 (1.0)**	**-2.4 (0.5)**	**-0.28 (0.5)**
FC3	**-3.04 (0.7)**	**-1.39 (0.7)**	**-2.17 (0.9)**	**-0.41 (0.9)**	**-2.27 (0.7)**	**-0.31 (0.7)**
FC4	**-3.27 (0.8)**	**-1.37 (0.8)**	**-2.35 (1.0)**	**0.07 (1.0)**	**-2.36 (0.5)**	**-0.36 (0.5)**
FC5	**-2.68 (0.9)**	**-1.28 (0.9)**	**-1.94 (0.9)**	**-0.31 (0.9)**	**-2.03 (0.9)**	**-0.16 (0.9)**
FC6	**-3.0 (0.9)**	**-1.25 (0.9)**	**-2.16 (1.0)**	**-0.19 (1.0)**	**-2.1 (0.4)**	**-0.22 (0.4)**
Cz	**-2.84 (0.8)**	**-1.17 (0.8)**	**-1.95 (1.0)**	**-0.07 (1.0)**	**-2.1 (0.5)**	**-0.35 (0.5)**
C1	**-2.91 (0.8)**	**-1.13 (0.8)**	**-1.97 (1.0)**	**-0.29 (1.0)**	**-2.03 (0.5)**	**-0.38 (0.5)**
C2	**-2.8 (0.7)**	**-1.22 (0.7)**	**-1.98 (1.0)**	**-0.1 (1.0)**	**-2.17 (0.6)**	**-0.38 (0.6)**
C3	**-2.73 (0.9)**	**-1.21 (0.9)**	**-1.95 (1.0)**	**-0.37 (1.0)**	**-2.02 (0.7)**	**-0.41 (0.7)**
C4	**-2.81 (0.6)**	**-1.09 (0.6)**	**-1.96 (1.0)**	**0.02 (1.0)**	**-2.07 (0.6)**	**-0.21 (0.6)**
C5	**-2.28 (0.8)**	**-1.09 (0.8)**	**-1.67 (0.9)**	**-0.36 (0.9)**	**-1.7 (0.7)**	**-0.28 (0.7)**
C6	**-2.34 (0.7)**	**-1.0 (0.7)**	**-1.64 (1.0)**	**0.09 (1.0)**	**-1.8 (0.6)**	**-0.2 (0.6)**

Note 1: means with standard deviations in parentheses unless otherwise specified. Note 2: More negative values indicate larger/healthier MMN amplitudes. Note 3: significant difference between the two cluster means (t-test, p-value < 0.05) on each channel was shown in bold.

**Table 2B:** Demographic, clinical, cognitive, functioning profiles of “Better” and “Poorer” clusters.

Variables	HC & Baseline FEP^a^		HC & Follow-Up FEP^b^		Baseline & Follow-Up FEP^c^	
	Cluster 1: Better functioning (N = 16)	Cluster 2: Poorer functioning (N = 37)	Cluster 1: Better functioning (N = 40)	Cluster 2: Poorer functioning (N = 11)	Cluster 1: Better functioning (N = 27)	Cluster 2: Poorer functioning (N = 11)
Patients (count, %)	5 (31.25%)	15 (40.54%)	12 (30.00%)	6 (54.55%)	N/A	N/A
Schizophrenia (count, %)	N/A	N/A	N/A	N/A	**8** **(29.63%)**	**8** **(72.73%)**
Age	24.44 (4.6)	22.14 (4.6)	23.08 (3.8)	23.09 (3.8)	22.67 (3.0)	23.91 (3.0)
Females (count, %)	6 (37.50%)	13 (35.14%)	14 (35.00%)	4 (36.36%)	10 (37.04%)	3 (27.27%)
Education (years)	**16.31** **(1.8)**	**14.89** **(1.8)**	15.38 (1.7)	15.36 (1.7)	14.89 (1.2)	15.27 (1.2)
UPSA	80.99 (9.8)	82.59 (9.8)	83.57 (9.0)	81.07 (9.0)	81.2 (12.8)	81.44 (12.8)
MCAS	52.21 (4.3)	51.88 (4.3)	**52.94** **(4.3)**	**49.22** **(4.3)**	49.33 (7.3)	44.6 (7.3)
MCAS Independent Subscore	9.75 (0.8)	9.31 (0.8)	**9.5** **(1.3)**	**8.0** **(1.3)**	**8.61** **(2.1)**	**6.78** **(2.1)**
MCAS Social Subscore	18.94 (2.0)	18.83 (2.0)	**19.39** **(1.6)**	**17.88** **(1.6)**	**17.91** **(3.4)**	**15.56** **(3.4)**
MATRICS Social Subscore	54.5 (8.7)	54.03 (8.7)	55.41 (8.2)	52.0 (8.2)	**57.0** **(14.2)**	**45.75** **(14.2)**
MATRICS Neurocognitive Composite Score	50.3 (5.1)	48.27 (5.1)	**50.66** **(5.0)**	**46.78** **(5.0)**	48.11 (9.3)	44.52 (9.3)
TASIT	55.25 (6.0)	54.87 (6.0)	**56.03** **(4.3)**	**52.62** **(4.3)**	54.87 (6.5)	52.12 (6.5)
PANSS Positive	N/A	N/A	N/A	N/A	13.33 (6.2)	15.3 (6.2)
PANSS Negative	N/A	N/A	N/A	N/A	11.15 (3.6)	12.6 (3.6)
PANSS General	N/A	N/A	N/A	N/A	27.81 (7.2)	31.5 (7.2)
PANSS Total	N/A	N/A	N/A	N/A	52.3 (15.6)	59.4 (15.6)

Note 1: means with standard deviations in parentheses. Note 2: Higher values indicate better functioning in UPSA/MCAS/MCAS-Independent/MCAS-Social/MATRICS-Social/MATRICS Neurocognitive/TASIT measures. Higher values indicate more symptomatic in PANSS Positive/PANSS Negative/PANSS General/PANSS Total measures. Note 3: ^a^Baseline measures for both HC and FEP subjects were used; ^b^Baseline measures were used for HC and follow-up measures were used for patients; ^c^Baseline measures were used for baseline patients and follow-up measures were used for follow-up patients. Note 4: Significant difference between the two cluster means (p-value < 0.05) was shown in bold.

We labeled the individuals in Cluster 1 as “Better functioning” and Cluster 2 as “Poorer functioning”, respectively. The demographic, clinical, cognitive, functioning profiles of these clusters are presented in **Table 2B**. Patient only clustering results were in **Supplementary Table S1**. Results of clustering among controls and patient baseline (**Table 2B**, left, **Supplementary Figure S3**) showed individuals in the “Better functioning” group, 31% of whom were patients and performed better but not significantly on all of the neurocognition, social cognition, and functioning measures, with an exception of UPSA task. Results of clustering among controls and follow-up patients (**Table 2B**, middle, **Supplementary Figure S4**) showed individuals in the “Better functioning” group, 30% of whom were patients and performed significantly better on all except two of the neurocognition, social cognition, and functioning measures. Results of clustering among baseline and follow-up patients (**Table 2B**, right) showed that patients in the “Better functioning” performed better on all the neurocognition, social cognition, and functioning measures, as well as had lower symptom severity scores (less symptomatic), and that group differences were significant on MCAS independent, MCAS social subscore, and MATRICS social subscore. 30% of patients in the “Better functioning” had SZ diagnosis.

### Clustering Analysis Using Changes in MMN Amplitude Over 6 Months

Results of patient’s MMN change over 6-months across 24 frontocentral channels were shown in **Table 3A**. Consistently across all 24 channels, Cluster 1 patients had, on average, bigger MMN amplitudes at follow-up than their baseline MMN, resulting in more negative MMN changes. In contrast, patients in the Cluster 2 group had smaller MMN at follow-up than their baseline amplitude, resulting in more positive MMN change. In each channel except FC5 site, differences between clusters 1 and 2 were significant at p < 0.05 (**Table 3A**). These results indicated that patients in Cluster 1 as a group had improved MMN over time whereas patients in Cluster 2 had worsening MMN responses. The demographic, clinical, cognitive, functioning profiles of “better” and “poorer” clusters are presented in **Table 3B**. Patients in the “Better functioning” cluster performed better on all the neurocognition, social cognition, and functioning measures, as well as had lower symptom severity scores than those in the “Poorer functioning” group. However, differences in each cognitive or functioning measure were not statistically significant except for the MCAS social subscore.

Table 3Clustering results of MMN change over 6-months and the clinical, cognitive, and functional characterizations of clusters.

**Table 3A:** Clustering results of MMN change over 6-months across 24 frontocentral channels.

Electrode sites	FEP (Change in MMN)	
	Cluster 1: Better functioning(N = 10)	Cluster 2: Poorer functioning(N = 8)
AFz	**-0.79 (1.8)**	**2.34 (1.8)**
AF3	**-1.06 (1.2)**	**1.8 (1.2)**
AF4	**-0.81 (1.7)**	**2.09 (1.7)**
Fz	**-0.65 (1.6)**	**1.39 (1.6)**
F1	**-0.87 (1.4)**	**1.62 (1.4)**
F2	**-0.69 (1.3)**	**1.65 (1.3)**
F3	**-0.84 (1.3)**	**1.54 (1.3)**
F4	**-1.27 (1.1)**	**1.45 (1.1)**
F5	**-0.74 (1.6)**	**1.53 (1.6)**
F6	**-1.31 (1.1)**	**1.79 (1.1)**
FCz	**-0.48 (1.2)**	**1.38 (1.2)**
FC1	**-0.55 (1.2)**	**1.33 (1.2)**
FC2	**-0.52 (1.2)**	**1.39 (1.2)**
FC3	**-0.42 (1.2)**	**1.13 (1.2)**
FC4	**-0.47 (0.8)**	**1.29 (0.8)**
FC5	-0.25 (1.3)	1.05 (1.3)
FC6	**-0.87 (0.8)**	**1.21 (0.8)**
Cz	**-0.39 (0.9)**	**1.1 (0.9)**
C1	**-0.63 (0.9)**	**1.0 (0.9)**
C2	**-0.4 (0.8)**	**0.99 (0.8)**
C3	**-0.5 (1.0)**	**0.95 (1.0)**
C4	**-0.53 (0.8)**	**1.04 (0.8)**
C5	**-0.37 (1.1)**	**0.73 (1.1)**
C6	**-0.41 (0.6)**	**1.15 (0.6)**

Note 1: means with standard deviations in parentheses. Note 2: In each channel, significant difference between the two cluster means (t-test, p-value < 0.05) was shown in bold.

**Table 3B:** Demographic, clinical, cognitive, functioning profiles of “Better” and “Poorer” clusters.

Variables	FEP (Change in MMN)^d^	
	Cluster 1: Better functioning (N = 10)	Cluster 2: Poorer functioning (N = 8)
Schizophrenia (count, %)	5 (50.00%)	3 (37.50%)
Age	23.4 (3.5)	23.38 (3.5)
Females (count,%)	4 (40.00%)	2 (25.00%)
Education (years)	14.9 (1.8)	15.25 (1.8)
UPSA	88.23 (14.6)	75.19 (14.6)
MCAS	49.33 (5.7)	46.5 (5.7)
MCAS Independent Subscore	**8.0 (1.5)**	**6.5 (1.5)**
MCAS Social Subscore	17.67 (2.1)	16.67 (2.1)
MATRICS Social Subscore	59.0 (16.1)	51.14 (16.1)
MATRICS Neurocognitive Composite Score	50.0 (10.6)	46.81 (10.6)
TASIT	55.89 (5.7)	52.83 (5.7)
PANSS Positive	12.0 (5.3)	14.5 (5.3)
PANSS Negative	10.33 (3.7)	10.5 (3.7)
PANSS General	23.56 (8.2)	30.25 (8.2)
PANSS Total	45.89 (15.9)	55.25 (15.9)

Note 1: means with standard deviations in parentheses unless otherwise specified. Note 2: ^d^Individual’s follow-up measures were used.

### The Magnitude of Delta, Theta, Alpha Frequency Activity in Controls and Patients

**Figure 5:**
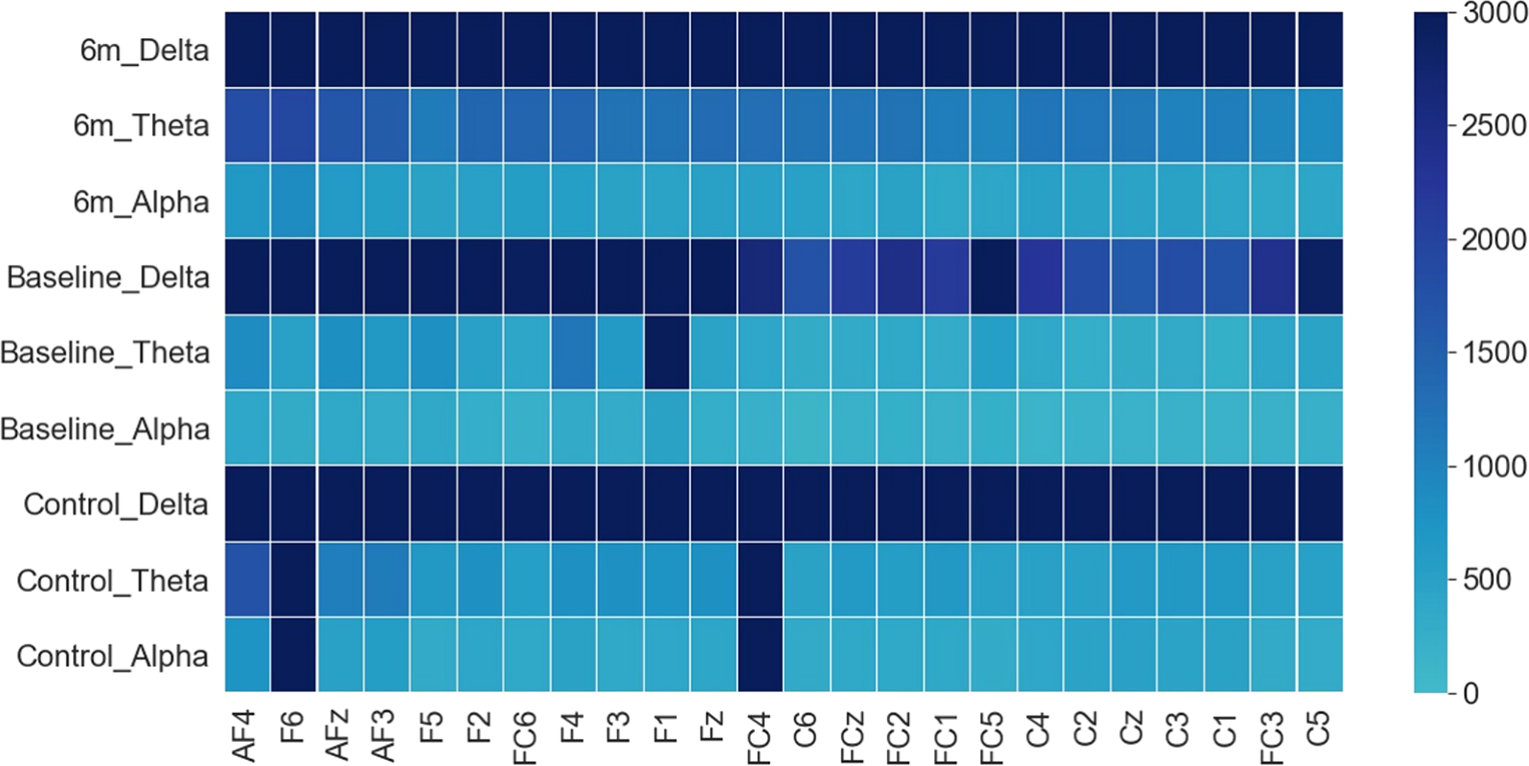
Grand average of AverageDifference across Delta, Theta and Alpha frequency bands in HC (bottom three rows), FEP baseline (middle three rows), and FEP follow-up (top three rows). X axis: electrode sites; Y axis (far right): the absolute value of the average AverageDifference. In all groups, the delta frequency showed the strongest signal across all 24 channels.

The overall magnitudes of the AverageDifference indices across three frequency bands were presented in **Figure 5**. Results showed that in both controls and patients the magnitudes of AverageDifference at delta frequency were statistically significantly higher than theta or alpha frequencies across most of the 24 channels (**Figure 5**). In controls p-value differences between delta and theta and between delta and alpha were *p* =1.1E-28 and *p* = 1.8E-22, respectively; in patient’s baseline, p-value differences were *p* =1.7E-21 and *p* = 1.1E-05, respectively. Thus, clustering analyses were only performed using AverageDifference index on delta frequency.

### Clustering Analysis Using AverageDifference on Delta Frequency Measure

**Table 4A** showed results using AverageDifference index on delta frequency over 24 frontocentral channels. In all three sets of clustering analyses (HC & baseline FEP; HC & follow-up FEP; baseline & follow-up FEP), the AverageDifference values across all 24 channels were greater in Cluster 1 than in Cluster 2, indicating that individuals as a group in Cluster 1 had AvgS2 > AvgS1 response, resulting in positive AverageDifference values, whereas individuals in Cluster 2 had AvgS2 < AvgS1 values, resulting in negative AverageDifference.

Table 4Clustering results of AverageDifference index and the clinical, cognitive, and functional characterizations of clusters.

**Table 4A:** Clusters results of AverageDifference across 24 frontocentral channels.

Electrodesites	HC & Baseline FEP		HC & Follow-Up FEP		Baseline & Follow-Up FEP	
	Cluster 1: higher AverageDifference(N = 29)	Cluster 2: lower AverageDifference(N = 22)	Cluster 1: higher AverageDifference(N = 24)	Cluster 2: lower AverageDifference(N = 23)	Cluster 1: higher AverageDifference(N = 10)	Cluster 2: lower AverageDifference(N = 26)
AFz	**3.2 (3.9)**	**-6.4 (3.9)**	**3.3(3.6)**	**-6.0(3.6)**	**5.5(3.1)**	**-3.5 (3.1)**
AF3	**3.4 (3.8)**	**-5.7 (3.7)**	**3.6 (3.4)**	**-5.5(3.4)**	**6.0(2.3)**	**-3.2 (2.3)**
AF4	**3.3(4.5)**	**-6.2(4.5)**	**3.8(4.0)**	**-6.3(4.0)**	**5.6 (4.1)**	**-3.6 (4.1)**
Fz	**1.8(2.0)**	**-3.2(2.0)**	**2.0(1.8)**	**-3.1 (1.9)**	**2.7 (1.4)**	**-1.6 (1.4)**
F1	**2.2(2.2)**	**-3.2 (2.2)**	**1.9 (1.6)**	**-3.2 (1.1)**	**2.7 (1.4)**	**-1.2(1.4)**
F2	**1.6 (2.3)**	**-3.1 (2.3)**	**2.0 (1.8)**	**-3.0 (1.8)**	**2.8 (1.6)**	**-1.9 (1.6)**
F3	**1.6 (2.3)**	**-3.3 (2.3)**	**1.9 (1.6)**	**-3.1 (1.7)**	**2.9 (1.3)**	**-1.7 (1.3)**
F4	**2.9(5.9)**	**-2.9 (5.9)**	**1.9(1.9)**	**-3.0 (1.9)**	**3.0 (1.9)**	**-0.04 (1.9)**
F5	**1.6 (2.8)**	**-3.9 (2.8)**	**2.2 (2.7)**	**-3.6 (2.7)**	**3.4(2.5)**	**-1.6 (2.5)**
F6	**1.9 (3.2)**	**-4.5 (3.2)**	**1.7 (3.3)**	**-4.7 (3.3)**	**3.5(3.6)**	**-2.1 (3.6)**
FCz	**1.1 (1.5)**	**-2.0 (1.5)**	**1.3 (1.4)**	**-2.1 (1.4)**	**1.4 (0.8)**	**-0.9 (0.8)**
FC1	**1.4 (1.6)**	**-1.9 (1.6)**	**1.4(1.6)**	**-1.8 (1.6)**	**1.5 (0.7)**	**-0.9 (0.7)**
FC2	**1.0 (1.6)**	**-1.8 (1.6)**	**1.2(1.4)**	**-1.8 (1.4)**	**1.6(0.8)**	**-1.1 (0.8)**
FC3	**0.9 (1.6)**	**-2.2 (1.6)**	**1.2 (1.3)**	**-2.1 (1.3)**	**1.3 (1.3)**	**-0.8 (1.3)**
FC4	**2.9 (9.9)**	**-1.5 (9.9)**	**3.4 (11)**	**-1.6 (11)**	**1.7 (0.9)**	**-1.1 (0.9)**
FC5	**0.9 (2.4)**	**-2.6 (2.4)**	**1.1 (1.5)**	**-2.4 (1.5)**	**1.3 (1.6)**	**-0.6 (1.6)**
FC6	**0.9(1.9)**	**-1.6 (1.9)**	**1.0(1.5)**	**-1.8 (1.5)**	**1.6 (1.6)**	**-1.3 (1.6)**
Cz	**0.7 (1.6)**	**-1.3 (1.6)**	**0.8 (1.6)**	**-1.5 (1.6)**	**0.9 (0.5)**	**-0.7 (0.5)**
C1	**1.0 (1.9)**	**-1.1 (1.9)**	**1.2 (1.9)**	**-1.3 (1.9)**	**0.9 (0.4)**	**-0.6 (0.4)**
C2	**0.7 (1.5)**	**-1.1 (1.5)**	**0.8 (1.3)**	**-1.3 (1.3)**	**1.0(0.5)**	**-0.6 (0.5)**
C3	**0.6 (1.3)**	**-1.2 (1.3)**	**0.8 (1.1)**	**-1.2 (1.1)**	**0.7 (0.5)**	**-0.5 (0.5)**
C4	**0.6 (1.8)**	**-0.9 (1.8)**	**0.8 (1.3)**	**-1.1 (1.3)**	**1.2 (0.6)**	**-0.9 (0.6)**
C5	**0.6 (1.4)**	**-1.5 (1.4)**	**0.9(1.0)**	**-1.5 (1.0)**	**0.6 (0.7)**	**-0.5 (0.7)**
C6	**0.6 (1.3)**	**-0.8 (1.3)**	**0.6 (1.1)**	**-1.0 (1.1)**	**0.9 (0.9)**	**-0.6 (0.9)**

Note 1: means with standard deviations in parentheses. Note 2: In each channel, significant difference between the two cluster means (t-test, p-value < 0.05) was shown in bold. Note 3: In each cell, AverageDifference and standard deviations denote E5 (10^5).

**Table 4B:** Demographic, clinical, cognitive, functioning profiles of two clusters.

Variables	HC & Baseline FEP^a^		HC & Follow-Up FEP^b^		Baseline & Follow-Up FEP^c^	
	Cluster 1: higher AverageDifference (N = 29)	Cluster 2: lower AverageDifference(N = 22)	Cluster 1: higher AverageDifference(N = 24)	Cluster 2: lower AverageDifference(N = 23)	Cluster 1: higher AverageDifference(N = 10)	Cluster 2: lower AverageDifference(N = 26)
Patients (count, %)	12 (41.38%)	8 (36.36%)	7 (29.17%)	9 (39.13%)	N/A	N/A
Schizophrenia (count, %)	N/A	N/A	N/A	N/A	6 (60.00%)	10 (38.46%)
Age	22.9 (2.8)	22.82 (2.8)	22.92 (2.8)	23.39 (2.8)	23.0 (3.0)	23.08 (3.0)
Females (count, %)	7 (24.14%)	11 (50.00%)	6 (25.00%)	10 (43.48%)	2 (20.00%)	10 (38.46%)
Education (years)	15.24 (1.6)	15.36 (1.6)	15.12 (1.5)	15.7 (1.5)	14.6 (0.8)	15.23 (0.8)
UPSA	81.7 (9.0)	82.07 (9.0)	79.79 (12.2)	85.47 (12.2)	76.46 (11.9)	82.44 (11.9)
MCAS	52.3 (4.7)	51.4 (4.7)	52.48 (4.5)	52.57 (4.5)	48.89 (5.3)	48.08 (5.3)
MCAS Independent Subscore	9.55 (1.0)	9.27 (1.0)	9.5 (1.3)	9.19 (1.3)	8.38 (1.8)	8.14 (1.8)
MCAS Social Subscore	18.9 (2.3)	18.77 (2.3)	19.1 (1.7)	19.33 (1.7)	17.62 (2.5)	17.18 (2.5)
MATRICS Social Subscore	53.71 (9.7)	54.64 (9.7)	54.32 (9.1)	54.05 (9.1)	51.14 (12.9)	54.24 (12.9)
MATRICS Neurocognitive Composite Score	47.63 (6.5)	50.22 (6.5)	49.45 (5.7)	50.72 (5.7)	46.07 (5.4)	47.79 (5.4)
TASIT	53.65 (5.1)	56.29 (5.1)	**53.67** **(4.5)**	**56.47** **(4.5)**	52.43 (5.3)	54.5 (5.3)
PANSS Positive	N/A	N/A	N/A	N/A	12.44 (5.7)	14.08 (5.7)
PANSS Negative	N/A	N/A	N/A	N/A	10.67 (4.2)	11.77 (4.2)
PANSS General	N/A	N/A	N/A	N/A	28.11 (7.7)	29.0 (7.7)
PANSS Total	N/A	N/A	N/A	N/A	51.22 (16.7)	54.85 (16.7)

Note 1: means with standard deviations in parentheses unless otherwise specified. Note 2: ^a^Baseline measures were used for both HC and FEP subjects; ^b^Baseline measures were used for HC and follow-up measures were used for patients; ^c^Baseline measures were used for baseline patients and follow-up measures were used for follow-up patients.

We labeled Cluster 1 as “higher AverageDifference” and Cluster 2 as “lower AverageDifference”, respectively. The demographic, clinical, cognitive, functioning profiles of these clusters were presented in **Table 4B**. Patient only clustering results were in **Supplementary Table S2**. Results of the clustering among controls and baseline patients (**Table 4B**, left) showed that 41% of individuals in the “higher AverageDifference” group were patients. Individuals in the “higher AverageDifference” performed better on MCAS functioning measures (total score, independent subscore, and social subscore) than those in the “lower AverageDifference” but not on neurocognition, social cognition, or UPSA functioning tasks. Among controls and follow-up patients (**Table 4B**, middle), results showed that 29% in the “higher AverageDifference” cluster were patients. Individuals in the “higher AverageDifference” performed better on MCAS independent subscore and MATRICS social subscore, but worse on neurocognition, TASIT, and other functioning measures. Results of clustering among baseline and follow-up patients (**Table 4B**, right) showed patients in the “higher AverageDifference” cluster performed better on MCAS measures (total score, independent subscore, and social subscore) and had lower symptom severity compared to those in the “lower AverageDifference” group but not on all the other measures (UPSA, neurocognition, MATRICS social, and TASIT). None was statistically significant at p < 0.05.

## Discussion

In this study, we used an unsupervised ML k-means algorithm to empirically stratify individuals into more homogeneous subgroups on the basis of multi-channel MMN data in a sample of controls and patients with FEP. We then characterized the functional, cognitive, and clinical profiles of these neurobiologically derived subgroups. We found, firstly, a consistent pattern of two distinctive subgroups across 24 frontocentral channels. Secondly, the two subgroups derived from MMN amplitude could be mapped onto either better or poorer clinical, cognitive, and functioning profiles. Thirdly, we examined the longitudinal MMN change over time and identified two subgroups of patients, one who showed improved MMN overtime and one who showed worsening of MMN overtime. Patients with improved MMN also had better follow-up clinical, cognitive, and functioning profile than those with worsening MMN. Fourthly, among the low frequency in the delta, theta, and alpha frequency bands, delta frequency appeared to be mostly relevant to the observed S1 and S2 EEG responses in both controls and patients. Finally, although delta frequency AverageDifference index could also empirically produce two distinctive subgroups, individuals in the higher AverageDifference cluster were not necessarily associated with better clinical, cognitive, and functioning profile than those in the lower AverageDifference group. To our knowledge, our study is the first to link neurophysiological derived subgroups with distinct cognitive and functional outcome characteristics. In addition, we demonstrated that variability in MMN change overtime is associated with symptom and functional outcomes.

### Heterogeneity in Patients and Controls

Heterogeneity is a major barrier to understand disease mechanisms and identify individuals with different recovery trajectories. As **Figure 4** shows, the EEG data of our sample clearly indicates that there exists a large variability not only among patients but also among controls. In addition, when both controls and patients were included in the MMN clustering analyses, 30% of patients were classified in the “better functioning” group. The combination of clustering techniques and multi-channel MMN activity employed in the current study facilitates the identification of neurobiological homogeneous subgroups. In two prior studies, we have used K-means analyses and identified distinct “Bio-classes” (38) among patients and controls and unique functional trajectories (4) among FEP patients that do not respect clinical diagnosis boundaries. Within each class, individuals shared a similar neurobiological profile or functional outcome trajectories that uniquely distinguished among the groups. These studies present a diagnosis-free approach to integrate information across biomarkers, yielding neurobiologically distinct subgroups and providing strong evidence supporting the superiority of neurobiological vs. clinical classification in differentiating psychotic disorders.

The presence of distinct neurobiological profiles among controls and patients brings into question the appropriateness of using diagnosis based on patient–control comparison analysis in MMN research, particularly during the early stage of illness. The relatively small effect size of dMMN deficit (20) reported in the meta-analyses is consistent with the notion of a large variability among FEP patients. The two subgroups identified in each of the three clustering models using 24-channel MMN are highly consistent and concordant in terms of the overall sample characteristics. MMN across 24 channels was consistently and significantly bigger in one subgroup over the other (**Table 2A**). We labeled the larger MMN subgroup as “Better functioning” as individuals in this group were consistently showing an overall pattern of better cognitive, social cognition, and functioning performances than the “poor functioning” individuals, regardless of whether controls were included in the models or not. That is, although significant differences were not observed in some clinical, cognitive, and functioning variables, the overall “gestalt” pattern was consistent. These results suggested that the ML data driven approach is a useful strategy to reduce heterogeneity and provide insight into clinically meaningful subgroups of a cohort. Also, results among baseline and follow-up patient clustering (**Table 2B**, right) showed that, patients in the “Better functioning” performed better on all the neurocognition, social cognition, and functioning measures, as well as had lower symptom severity scores (less symptomatic). Among patients in the “Better functioning”, 30% had SZ diagnosis. This result is in line with the literature that patients with SZ generally exhibit more impairment and greater symptom severity compared to patients with affective psychosis. One prior study (35) has found that the most impaired baseline MMN amplitudes corresponded to the most severe functional disability at follow-up, consistent with the findings obtain in our study.

### Longitudinal MMN Change Is Associated With Better Clinical and Outcome Characteristics

Results of our analyses showed that MMN changes across 24 channels were significantly improved in one subgroup over the other and that patients with improved MMN over the 6-months follow-up period also had better follow-up clinical, cognitive, and functioning profile than those with worsening MMN (**Tables 3A, B** **)**. It is striking that we observed again a consistent overall “gestalt” pattern that patients in the “Better functioning” cluster performed better on all the neurocognition, social cognition, and functioning measures, as well as had lower symptom severity scores than those in the “Poorer functioning” group. Although differences in most cognitive and functioning variables were largely non-significant, this is likely due to small sample size in each of the clusters (Ns = 10 and 8). Nonetheless, the overall pattern is consistent and among the variables examined, the UPSA, MATRICS social, and PANSS total had the biggest mean differences. These results support the notion that the ML data driven approach is useful to explore heterogeneity and facilitate the identification of neurobiological homogeneous subgroups. Koshiyama et al. (56) reported that MMN of FEP patients do not change significantly over time, while (36) observed that MMN deteriorated in patients over time. These prior reports also indicate heterogeneity in MMN change over time among FEP patients. Our present findings suggest that combining ML and follow-up clinical characterization approaches can potentially identify individuals at greater risk of poorer functional outcomes at a “critical period” of neuronal and psychosocial plasticity and for whom there is a “window of opportunity” for treatment to achieve disproportionately favorable outcomes. These individuals can be targeted for earlier, more aggressive treatment interventions, both pharmacologically and psychosocial/cognitive intervention therapy, to reduce function deterioration and improve recovery.

### Significant Contribution of Delta Frequency During MMN

In this study, we explored the underlying EEG frequency responses during MMN by decomposing MMN data into low frequency range activity and calculated frequency specific “sum difference” (AverageDifference) indices to capture the overall differences between the averaged S2 and S1 segments. We found that the magnitudes of AverageDifference at delta frequency were significantly higher than those of theta or alpha frequencies across all 24 channels in controls and patients (**Figure 5**), suggesting that AverageDifference of delta frequency plays an important role in the MMN generation. Our results are consistent with Hong et al., 2012 who observed that delta range activities were found to explain the most variance of observed MMN abnormalities in SZ (41).

### Comparison of MMN and AverageDifference of Delta Frequency

As shown in **Table 4**, two distinct subgroups could also be consistently obtained in all three clustering models using the AverageDifference index of delta frequency, one cluster with “higher AverageDifference” values and the other with “lower AverageDifference”. While K-means generated a consistent pattern of two clusters using either multimodal MMN or low frequency measure, individuals in the higher AverageDifference cluster were not necessarily associated with better clinical, cognitive and functioning profile than those in the lower AverageDifference group. There are two major differences between MMN and the AverageDifference of delta frequency. MMN waveforms were generated directly by subtracting the average of S1 from the average of S2 waveforms between the time window of 120 to 250 ms. Although the same window was used for the AverageDifference, squared wavelet transformation is applied to each single trial response of the original S1 and S2 signals before computing the sum of the difference between averaged S1 and S2. In addition, AverageDifference index was derived using delta frequency only, which constitutes a subset of total MMN signals. As a result, the associated clinical and functioning variances were not fully captured in the AverageDifference of delta frequency. Our results suggest that MMN appeared to yield clinically meaningful interpretation, and MMN is superior to the AverageDifference index as a neurobiological measure for identifying clinically distinct subgroups and the application of squared wavelet transformation may not be an optimal method.

Our study has a number of limitations. Although several interesting and consistent findings were found, the study sample size is relatively small, particularly the patient’s longitudinal data. K-means is an exploratory research tool to discover new patterns. Although k-means clustering can sufficiently uncover patterns with a relatively small number of subjects (57, 58), supervised learning methods would be used, and a larger number of subjects in the future is needed to prove the results are generalizable for a larger population (59). Second, there are no follow-up data for controls, which limit our ability to determine the stability of cognitive, functioning, and EEG measures or typical changes that occur in all individuals. Third, because our focus was on deriving more homogeneous subgroups using agnostic approach on the basis of MMN or low frequency activities, we did not investigate the degree of consistence within patients by comparing PANSS-derived subgroups with MMN derived subgroups.

## Conclusions

In conclusion, the ML data-driven approach is a useful tool in FEP psychosis research to address heterogeneity and facilitate identification of clinically meaningful subgroups and patterns between MMN and clinical, cognitive, functioning characteristics.

## Data Availability Statement

The datasets generated for this study are available on request to the corresponding author.

## Ethics Statement

The studies involving human participants were reviewed and approved by Partners Human Research Committee. The patients/participants provided their written informed consent to participate in this study.

## Author Contributions

XQ, TH, and M-HH contributed to the conception and design of the study. AH organized the database. XQ, SL, and JT performed the statistical analysis. XQ wrote the first draft of the manuscript. M-HH, SL, and JT wrote sections of the manuscript. All authors contributed to the article and approved the submitted version.

## Funding

This publication was supported by funds received from National Institute of Mental Health grants NIMH [R01MH109687]: M-HH, PI.

## Conflict of Interest

The authors declare that the research was conducted in the absence of any commercial or financial relationships that could be construed as a potential conflict of interest.
