# Supplementary material for: Identifying Clinically and Functionally Distinct Groups Among Healthy Controls and First Episode Psychosis Patients by Clustering on EEG Patterns
Source: Front Psychiatry. 2020 Sep 18;11:541659. doi: 10.3389/fpsyt.2020.541659 (PMC7530247; doi:10.3389/fpsyt.2020.541659)

Supplementary Table S1: Patients only clustering results of MMN amplitude and characterizations of clusters

Table S1a: Clustering results of MMN over 24 frontocentral channels

| Electrode sites | Cluster 1: Better functioning  (N = 5) | Cluster 2:  Poorer functioning  (N = 15) | Cluster 1: Better functioning  (N = 12) | Cluster 2: Poorer functioning  (N = 6) |
| --- | --- | --- | --- | --- |
| AFz | -4.61 (1.0) | -2.17 (1.2) | -4.51 (1.1) | -1.48 (1.7) |
| AF3 | -4.97 (0.9) | -2.75 (1.1) | -4.79 (0.8) | -2.03 (1.2) |
| AF4 | -4.76 (0.8) | -2.53 (1.1) | -4.81 (0.8) | -1.95 (1.3) |
| Fz | -4.23 (1.3) | -2.12 (1.2) | -4.22 (1) | -1.59 (1.5) |
| F1 | -4.05 (0.6) | -2.34 (0.9) | -3.56 (0.9) | -1.88 (1.3) |
| F2 | -4.58 (0.5) | -2.76 (1) | -4.51 (0.7) | -2.17 (1.1) |
| F3 | -5.03 (0.6) | -2.9 (0.8) | -4.77 (0.7) | -2.29 (1.1) |
| F4 | -4.17 (0.5) | -2.64 (1) | -4.27 (1) | -2.29 (1) |
| F5 | -4 (0.6) | -2.71(0.9) | -4.21 (1.3) | -2.25 (1.1) |
| F6 | -3.24 (0.9) | -2.26(0.9) | -3.13 (1.2) | -1.93 (1.1) |
| FCz | -4.91 (1.1) | -2.85 (1.2) | -4.86 (0.8) | -1.62 (1.6) |
| FC1 | -4.86 (1) | -2.72 (1.4) | -4.52 (0.9) | -1.39 (1.6) |
| FC2 | -5 (1) | -2.86 (0.9) | -4.73 (0.9) | -2.14 (1.2) |
| FC3 | -5.05 (1) | -2.99 (0.9) | -4.89 (0.7) | -2.03 (1.3) |
| FC4 | -5.02 (1) | -2.77 (1) | -5 (1.1) | -2.19 (1.4) |
| FC5 | -4.4 (1.1) | -2.67 (1.1) | -4.43 (1.5) | -2.05 (1.6) |
| FC6 | -4.11 (0.9) | -2.6 (0.9) | -4.41 (0.9) | -1.85 (1) |
| Cz | -4.65 (0.7) | -3.01 (0.9) | -4.92 (0.8) | -2.1 (1.1) |
| C1 | -4.9 (0.9) | -2.91 (0.9) | -4.92 (0.6) | -2.11 (1.1) |
| C2 | -5.04 (0.7) | -2.96 (0.9) | -4.9 (0.6) | -2.11 (1.1) |
| C3 | -4.14 (0.8) | -2.82 (0.9) | -4.54 (0.6) | -2.07 (1) |
| C4 | -4.09 (0.6) | -2.83 (1.1) | -4.65 (1) | -2.13 (1) |
| C5 | -3.9 (0.5) | -2.76 (1) | -4.81 (1.1) | -1.98 (1.2) |
| C6 | -3.49 (0.7) | -2.46 (0.9) | -4.05 (1.1) | -1.48 (1.1) |

Note 1: means with standard deviations in parentheses unless otherwise specified. Note 2: More negative values indicate larger / healthier MMN amplitudes.

Supplementary Table S1b: Patient only demographic, clinical, cognitive, functioning profiles of “Better” and “Poorer” clusters

|  | Cluster 1: Better functioning  (N = 5) | Cluster 2:  Poorer functioning  (N = 15) | Cluster 1: Better functioning  (N = 12) | Cluster 2: Poorer functioning  (N = 6) |
| --- | --- | --- | --- | --- |
| Age | 24.0 (3.2) | 22.3 (3.1) | 23.2 (3.4) | 23.8 (3.5) |
| Females  (count, %) | 1 (20%) | 6 (40%) | 4 (33%) | 2 (33%) |
| Education (years) | 15.8 (1.8) | 14.7 (1.5) | 15.0 (1.6) | 15.2 (1.5) |
| UPSA | 73.4 (9.9) | 81.5 (11) | 83.9 (10.5) | 78.4 (17.0) |
| MCAS | 47.8 (4.5) | 48.2 (6.3) | 49.3 (5.9) | 44.8 (6.3) |
| MCAS Independent | 9.2 (1.3) | 8.4 (1.8) | 7.9 (1.9) | 6.0 (1.6) |
| MCAS Social | 17.0 (2.8) | 17.0 (3.2) | 17.8 (2.8) | 16.0 (2.2) |
| MATRICS Social | 53.4 (14.6) | 53.6 (10.8) | 57.5 (11.5) | 49.5 (19.6) |
| MATRICS Neurocognitive  Composite Score | 48.8 (7.2) | 45.2 (6.1) | 50.3 (6.1) | 44.4 (11.8) |
| TASIT | 55.3 (8.9) | 53.3 (6.0) | 56.1 (4.1) | 50.7 (6.6) |
| PANSS Positive | 18.8 (9.5) | 13 (5.2) | 12.3 (5.1) | 15.2 (6.4) |
| PANSS Negative | 13.2 (4.0) | 12.3 (3.9) | 9.8 (3.3) | 11.8 (4.2) |
| PANSS General | 32.2 (10.7) | 30.1 (7.1) | 25.2 (8.5) | 30.4 (7.7) |
| PANSS Total | 64.2 (21.3) | 55.3 (15.1) | 47.3 (15.9) | 57.4 (15.9) |

Means with standard deviations in parentheses. Note 2: Higher values indicate better functioning in UPSA / MCAS / MCAS-Independent / MCAS-Social / MATRICS-Social / MATRICS Neurocognitive / TASIT measures. Higher values indicate more symptomatic in PANSS Positive / PANSS Negative / PANSS General / PANSS Total measures.

Table S2: Patient only clustering results of AverageDifference index and characterizations of clusters

Table S2a: Clusters results of AverageDifference across 24 frontocentral channels

| Electrode sites | Cluster 1: higher AverageDifference  (N = 12) | Cluster 2: lower AverageDifference  (N = 8) | Cluster 1: higher AverageDifference  (N = 7) | Cluster 2: lower AverageDifference  (N = 9) |
| --- | --- | --- | --- | --- |
| AFz | 2.8 (4.1) | -4.8 (4.7) | 3 (3.3) | -4.5 (3.2) |
| AF3 | 2.3 (3) | -2.5 (1.2) | 1.5 (1.6) | -2.6 (0.8) |
| AF4 | 1.1 (2.9) | -2.5 (1.1) | 1.7 (1.4) | -2.2 (1.3) |
| Fz | 1.1 (3.1) | -2.7 (1.8) | 2.8 (3) | -2 (1.9) |
| F1 | 0.8 (3.3) | -1.5 (1.3) | 1.2 (1.2) | -1.1 (0.8) |
| F2 | 0.4 (1.6) | -1.3 (1) | 1 (0.8) | -1.1 (0.8) |
| F3 | 0.9 (1.2) | -1.4 (1.3) | 0.8 (0.9) | -1.3 (0.8) |
| F4 | 0.4 (1.3) | -0.6 (1.1) | 0.5 (0.6) | -1.1 (0.6) |
| F5 | 0.3 (1.2) | -0.8 (1) | 0.6 (0.4) | -0.9 (0.5) |
| F6 | 0.2 (1.7) | -0.7 (1) | 0.6 (0.6) | -0.9 (0.6) |
| FCz | 2.1 (5.1) | -4.7 (2.3) | 2.9 (4.1) | -5.1 (2.6) |
| FC1 | 2.7 (4.2) | -5.4 (3.6) | 2.7 (3.6) | -4.7 (2.6) |
| FC2 | 1.1 (2.2) | -2.4 (1) | 1.6 (1.8) | -2.2 (1.2) |
| FC3 | 0.9 (2.7) | -2.6 (1) | 1.5 (2) | -2.4 (1) |
| FC4 | 4 (9) | -2.4 (0.5) | 1.8 (2.3) | -2.5 (1.1) |
| FC5 | 1.7 (3.8) | -2.5 (0.7) | 0.8 (4.2) | -2.9 (2.3) |
| FC6 | 0.6 (2.2) | -1.5 (1) | 0.7 (1.1) | -2 (1.4) |
| Cz | 0.7 (1.8) | -1.3 (0.8) | 0.9 (1.2) | -1.6 (0.6) |
| C1 | 0.5 (1.9) | -1.4 (0.9) | 0.9 (1.1) | -1.4 (0.7) |
| C2 | 0.7 (1.3) | -1.1 (1.1) | 0.8 (1) | -1.5 (0.7) |
| C3 | 0.4 (1.1) | -0.6 (1.1) | 0.4 (0.8) | -1.3 (0.6) |
| C4 | 0.6 (1.7) | -0.7 (1) | 0.5 (0.8) | -1.2 (0.5) |
| C5 | 0.1 (2.3) | -0.7 (0.8) | 0.5 (0.9) | -1.1 (0.4) |
| C6 | 0.4 (1.4) | -0.7 (1) | 0.4 (0.5) | -1 (0.5) |

Note 1: means with standard deviations in parentheses. In each cell AverageDifference and standard deviations denotes E5 (10^5).

Table 2Sb: Patient only demographic, clinical, cognitive, functioning profiles of two clusters

|  | Cluster 1: higher AverageDifference  (N = 12) | Cluster 2: lower AverageDifference  (N = 8) | Cluster 1: higher AverageDifference  (N = 7) | Cluster 2: lower AverageDifference  (N = 9) |
| --- | --- | --- | --- | --- |
| Age | 22.7 (3.4) | 22.7 (3.1) | 22.6 (3.9) | 24.2 (3.0) |
| Females  (count, %) | 3 (25%) | 4 (50%) | 2 (29%) | 3 (33%) |
| Education (years) | 14.8 (1.8) | 15.1 (1.3) | 14.1 (1.1) | 16.0 (1.5) |
| UPSA | 81.7 (8.6) | 77.17 (14.5) | 75.7 (16.9) | 86.4 (6.4) |
| MCAS | 49.4 (5.9) | 46.1 (5.4) | 47.2 (5.7) | 49.4 (6.8) |
| MCAS Independent | 8.9 (1.4) | 8.1 (2.1) | 6.7 (1.5) | 7.7 (2.4) |
| MCAS Social | 17.8 (3.2) | 16.6 (2.8) | 15.7 (2.3) | 18.0 (2.6) |
| MATRICS Social | 53.1 (12.4) | 54.2 (10.9) | 55.0 (13.8) | 52.6 (14.9) |
| MATRICS Neurocognitive  Composite Score | 45.0 (6.7) | 47.9 (6.1) | 49.9 (5.2) | 48.9 (10.6) |
| TASIT | 52.5 (6.5) | 55.7 (6.0) | 51.4 (6.1) | 56.2 (4.6) |
| PANSS Positive | 12.4 (5.8) | 17.5 (7.3) | 13.8 (5.9) | 11.8 (5.5) |
| PANSS Negative | 11.7 (4.6) | 13.6 (1.9) | 11.3 (4.0) | 9.3 (3.0) |
| PANSS General | 29.7 (9.6) | 31.9 (4.5) | 29.3 (9.0) | 24.3 (8.8) |
| PANSS Total | 53.9 (19.3) | 63.0 (10.9) | 54.5 (18.0) | 45.4 (15.9) |

**Supplement Figure S1: Cluster configuration of MMN amplitude with the elbow method**


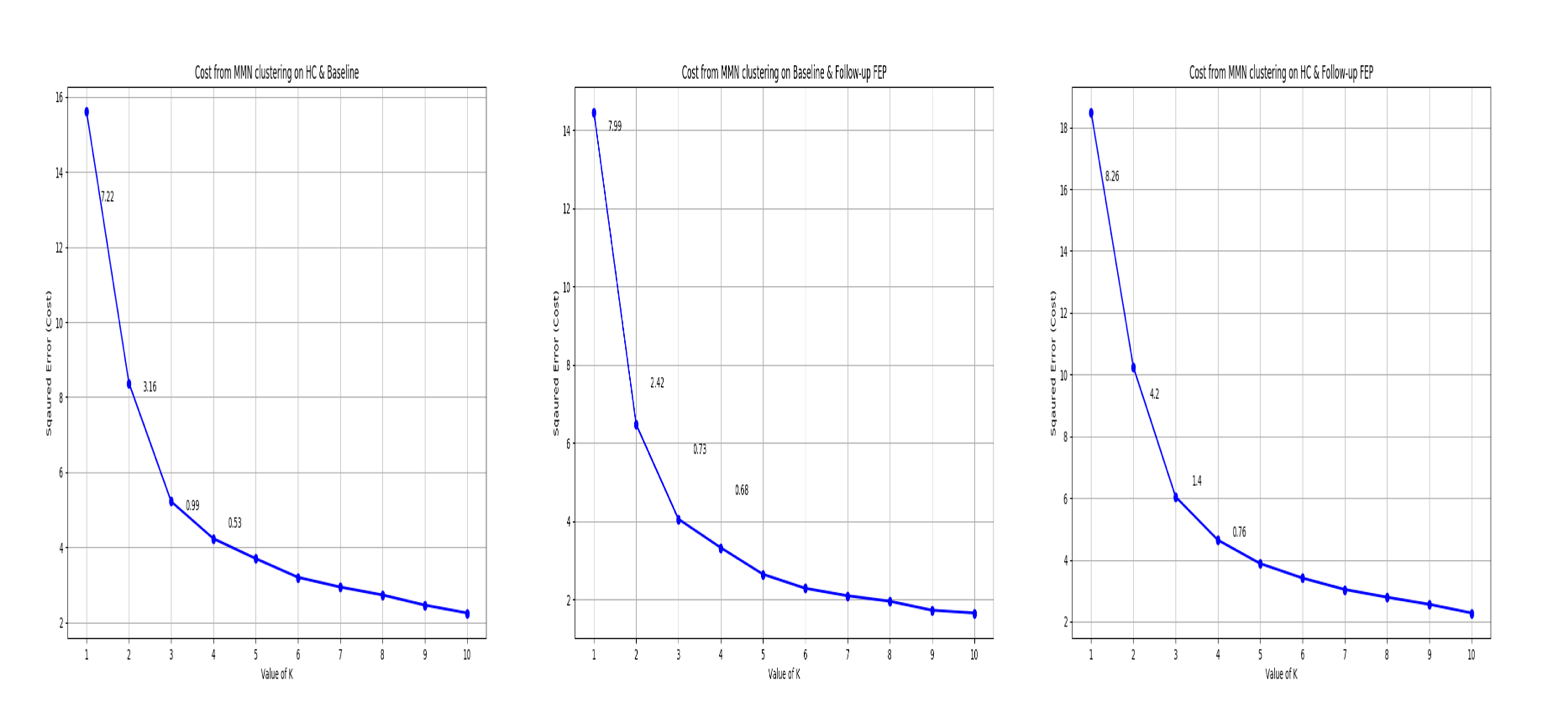


The highest drop of error/cost determines the best cluster configuration. In all three cases, two cluster configuration is shown to be the optimal number.

**Supplement Figure S2: Cluster configuration of AverageDifference with the elbow method**


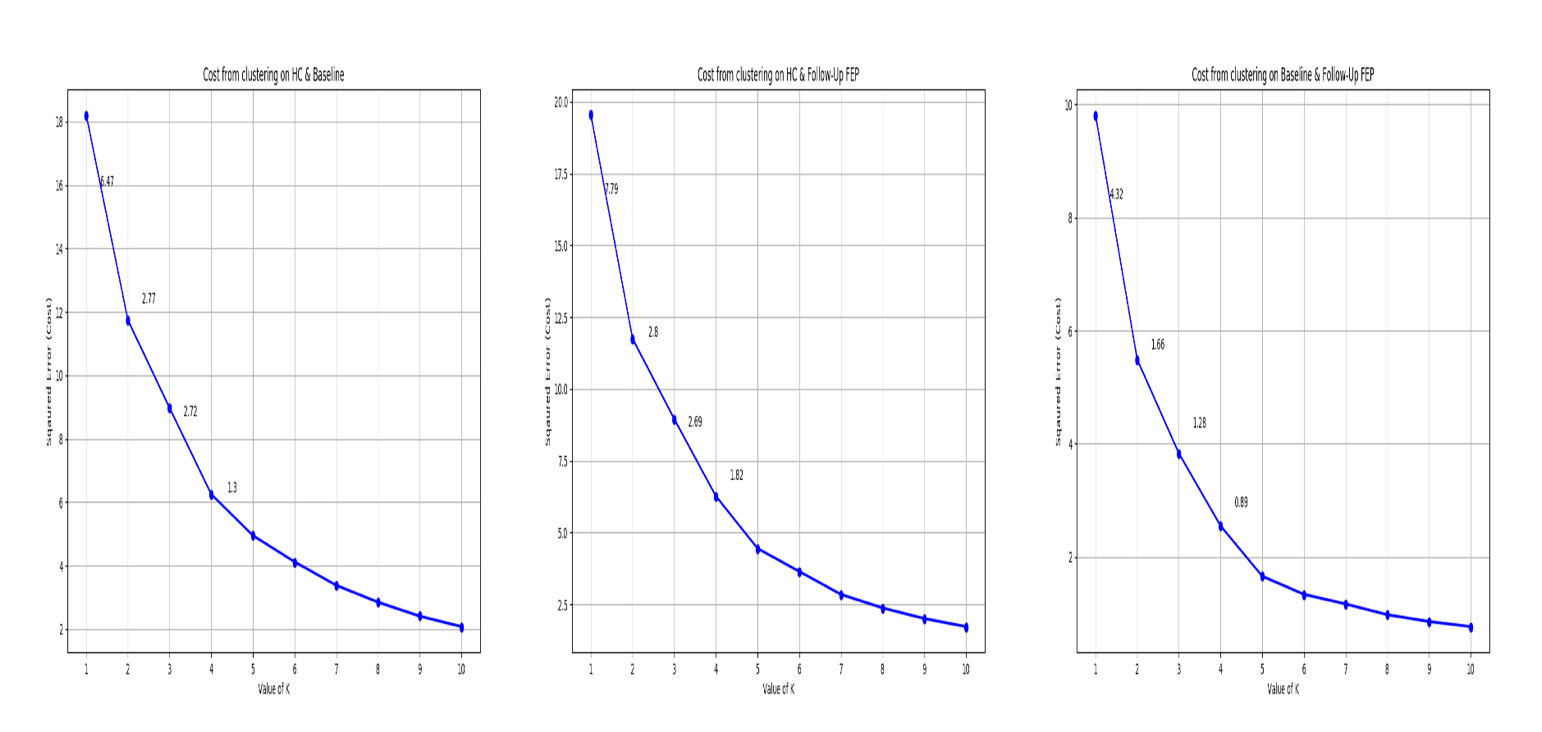


The highest drop of error/cost determines the best cluster configuration. In all three cases, two cluster configuration is shown to be the optimal number.

Figure S3. Grand averaged MMN of clustering 1 (better) and 2 (poorer) over 24 frontocentral channels (HC & Baseline FEP)


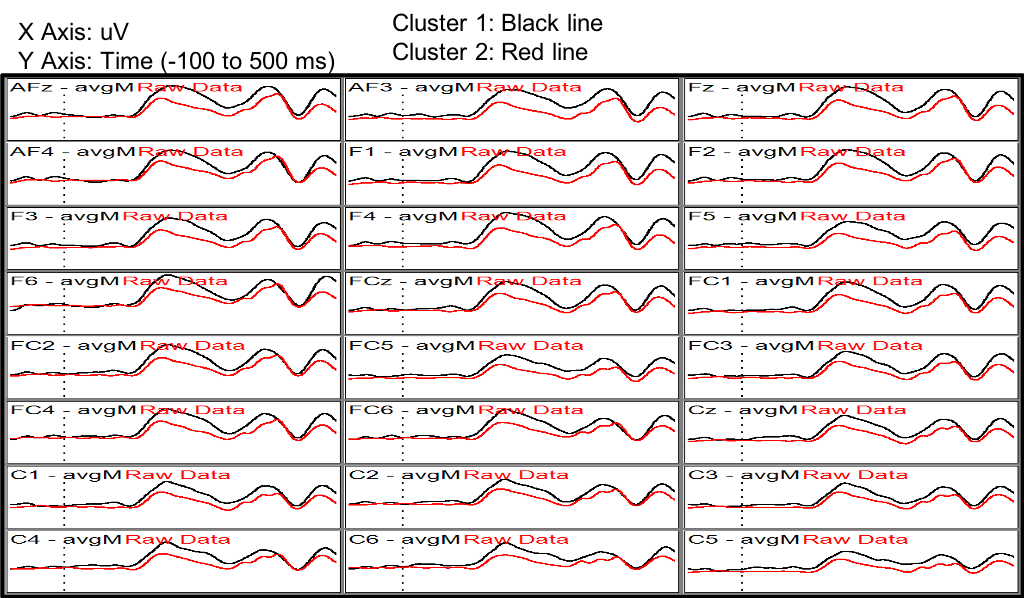


Figure S4. Grand averaged MMN in clustering 1 (better) and 2 (Poorer)over 24 frontocentral channels (HC & Follow-up FEP)


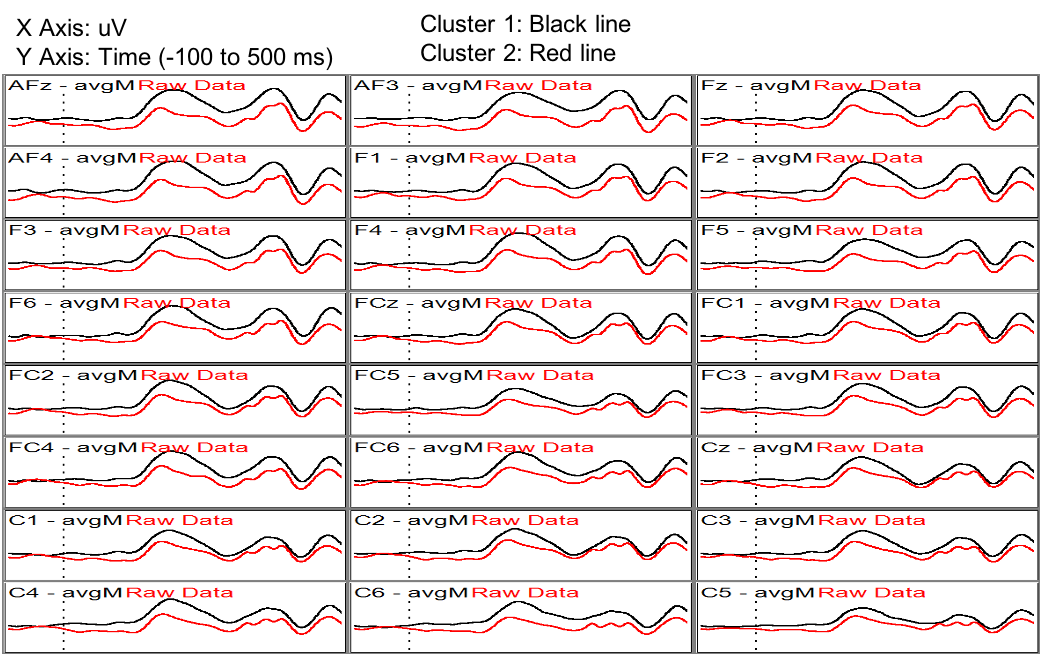

Supplement: Supplementary file 1 [file DataSheet_1.docx]
